# Hotspot and Frontier Analysis of Exercise Training Therapy for Heart Failure Complicated With Depression Based on Web of Science Database and Big Data Analysis

**DOI:** 10.3389/fcvm.2021.665993

**Published:** 2021-05-19

**Authors:** Yan Wang, Yuhong Jia, Molin Li, Sirui Jiao, Henan Zhao

**Affiliations:** ^1^Scientific Research Center, The Second Hospital of Dalian Medical University, Dalian, China; ^2^Department of Pathophysiology, College of Basic Medical Sciences, Dalian Medical University, Dalian, China; ^3^Department of Anatomy, College of Basic Medical Sciences, Dalian Medical University, Dalian, China; ^4^Graduate School of Dalian Medical University, Dalian, China

**Keywords:** heart failure, depression, exercise training, mitochondria, big data techniques

## Abstract

**Background:** Exercise training has been extensively studied in heart failure (HF) and psychological disorders, which has been shown to worsen each other. However, our understanding of how exercise simultaneously protect heart and brain of HF patients is still in its infancy. The purpose of this study was to take advantage of big data techniques to explore hotspots and frontiers of mechanisms that protect the heart and brain simultaneously through exercise training.

**Methods:** We studied the scientific publications on related research between January 1, 2003 to December 31, 2020 from the WoS Core Collection. Research hotspots were assessed through open-source software, CiteSpace, Pajek, and VOSviewer. Big data analysis and visualization were carried out using R, Cytoscape and Origin.

**Results:** From 2003 to 2020, the study on HF, depression, and exercise simultaneously was the lowest of all research sequences (two-way ANOVAs, *p* < 0.0001). Its linear regression coefficient *r* was 0.7641. The result of hotspot analysis of related keyword-driven research showed that inflammation and stress (including oxidative stress) were the common mechanisms. Through the further analyses, we noted that inflammation, stress, oxidative stress, apoptosis, reactive oxygen species, cell death, and the mechanisms related to mitochondrial biogenesis/homeostasis, could be regarded as the primary mechanism targets to study the simultaneous intervention of exercise on the heart and brain of HF patients with depression.

**Conclusions:** Our findings demonstrate the potential mechanism targets by which exercise interferes with both the heart and brain for HF patients with depression. We hope that they can boost the attention of other researchers and clinicians, and open up new avenues for designing more novel potential drugs to block heart-brain axis vicious circle.

## Introduction

In the field of cardiovascular disease (CVD) research, heart failure (HF) remains a rising global epidemic with an estimated prevalence of >37.7 million individuals globally (1), associated with significant mortality, morbidity, and health-care expenditures (2). Additionally, associated with a serious burden to patients, e.g., poor functioning and impaired health-related quality of life (QoL) and self-care, as well as frequent hospitalizations and high health care costs, anxiety and depression disorders are prevalent in patients with HF, especially chronic heart failure (CHF) (3–6). In a meta-analysis of eight studies examining the prospective associations between depressive symptoms or a depressive disorder and HF outcomes, Rutledge et al. (3) found that depressive symptoms or depressive disorders were highly prevalent risk factors for cardiac death or events in patients with established HF. In line with these clinical and experimental evidence, HF and depression have shown a worsening relationship with each other, just like a vicious cycle through the heart-brain axis (HBA) (7, 8).

Additionally, as a safe and effective therapy, moderate continuous training is recommended in the therapeutic approach to the stable HF patient, and is supported by the American Heart Association (AHA), the American College of Cardiology (ACC), and the Heart Failure Society of America at a Class I or II level (9, 10). On the other hand, there is also a general belief that physical activity and exercise have positive effects on mood and anxiety, and a great number of studies describe an association of physical activity and general well-being, mood and anxiety (11). In fact, it also appears that exercise training reduces depression in HF patients. A previous study reported that in coronary heart disease patients, aerobic exercise in a group setting appeared to have a similar impact on reducing depression to antidepressant medication (12). Blumenthal and colleagues demonstrated that in the HF-ACTION trial, the pre-specified combined end-point of death or hospitalization adjusted for baseline variables, was reduced by ~10% in the exercise group, and had significantly less depression at both 3 and 12 months (13). The role of exercise training and the mechanisms controlling have been extensively studied in CVD and psychological disorders, respectively, however, our understanding of how exercise improve outcome of HF complicated with depression is still in its infancy, especially with respect to interventions in the above-mentioned vicious circle. On the other hand, we have also noticed that some studies have combined these factors, but the new research area mainly focused on self-care, physical activity, outcomes, meta-analysis, etc. (14–16). Mechanism research is relatively weak, and interdisciplinary cooperation is also lacking. Therefore, we believe that relevant research is necessary, especially the potential mechanism targets.

Furthermore, since both the brain and heart are known to be with high energy demands, mitochondria have been identified and demonstrated to be key mediators in the pathogenesis and/or intervention of CVD and psychiatric disorders, respectively (17–20). It is now well-known that exercise can provide a potent stimulus in mitochondrial adaptations, which ultimately produce robust phenotypic changes in the quantity and quality of the organelle network, leading to greater health (21–24). Thus, this means that the information and knowledge generated through combining heart failure, depression, exercise and mitochondria can help us identify the critical mechanism of exercise therapy to prevent the vicious circle through HBA.

The other thing needed to be mentioned is new data processing techniques, which often involves big data analysis and its highly dependent data visualization (11). Big data techniques can offer an algorithm to get more information because of arbitrary choices in data construction and give pointers to the most important choices in the resulting mechanism. Simultaneously, big data analysis based on publications representing the wisdom of human beings can effectively avoid the limitation in the analyses of the findings that may be induced by the subjective choice of some researchers. Therefore, in this work, we will take advantage of big data techniques (analysis and visualization) and publication database to explore the hotspots and frontiers of related mechanisms that protect the heart and brain simultaneously through exercise training. In addition, we hope to reveal the crosstalk between mitochondrial dysfunction and the above research hotspots, provide more new ideas for development of exercise therapy, and open up new avenues for designing more novel potential drugs for HF patients, especially complicated with depression, and those who cannot perform exercise training.

## Methods

### Data Source

The Web of Science (WoS) Core Collection is the most frequently used citation database for scientometric analysis (25, 26). Therefore, in this study, in order to the greatest extent to achieve the goals of the study, we adopted the approach of using a complete dataset of scientific publications on related research between January 1, 2003 to December 31, 2020 from the WoS Core Collection as the basis for constructing the scientometric networks of disciplines.

### Data Analysis and Visualization

To assess any weight changes of research hotspots, degree, weighted degree, betweenness centrality and eigenvector centrality were calculated through open-source software, CiteSpace (v5.7.R3), Pajek (v5.09), and VOSviewer (v1.6.15). Big data analysis and visualization were carried out using R software (v4.0.2), Cytoscape (v3.8.2) and Origin software (v2021b). Principal component analysis (PCA), an algorithm for the group heterogeneity through reconstructing the reprogramming zone, was performed using the FactoMineR package of the R software.

### Search Strategy

All electronic searches were conducted on a single day, December 31, 2020, to avoid changes in citation rate as much as possible. This search included articles, reviews, proceedings, meeting abstracts, and letters. Any overlap between articles found across the different source materials were removed by CiteSpace. The following keywords were used in searches in the Title/Abstract/Keywords of each article in the respective databases: “heart failure,” “depression,” “exercise,” “inflammation,” “oxidative stress,” and “mitochondria.” The txt data downloaded from WoS were imported into Microsoft Excel 365 first. Further, the title, authors, countries, institutions, abstract, and keywords of each article were analyzed.

### Statistical Analysis

OriginPro 2021 (OriginLab Corporation) was used to analyze the time trend of the publications. The logistic regression model, *f* (x) = [a+bx], was used to calculate the cumulative volume and to predict future trend of papers in this field. VOSviewer, Pajek and CiteSpace were used to analyze the co-occurrence network. Two-way ANOVAs was performed by GraphPad Prism (v5.0). Statistical significance was established a priori as *p* < 0.05.

## Results

### Characteristic Analysis of Related Publications (Number and Trends) in Time

**Figure 1:**
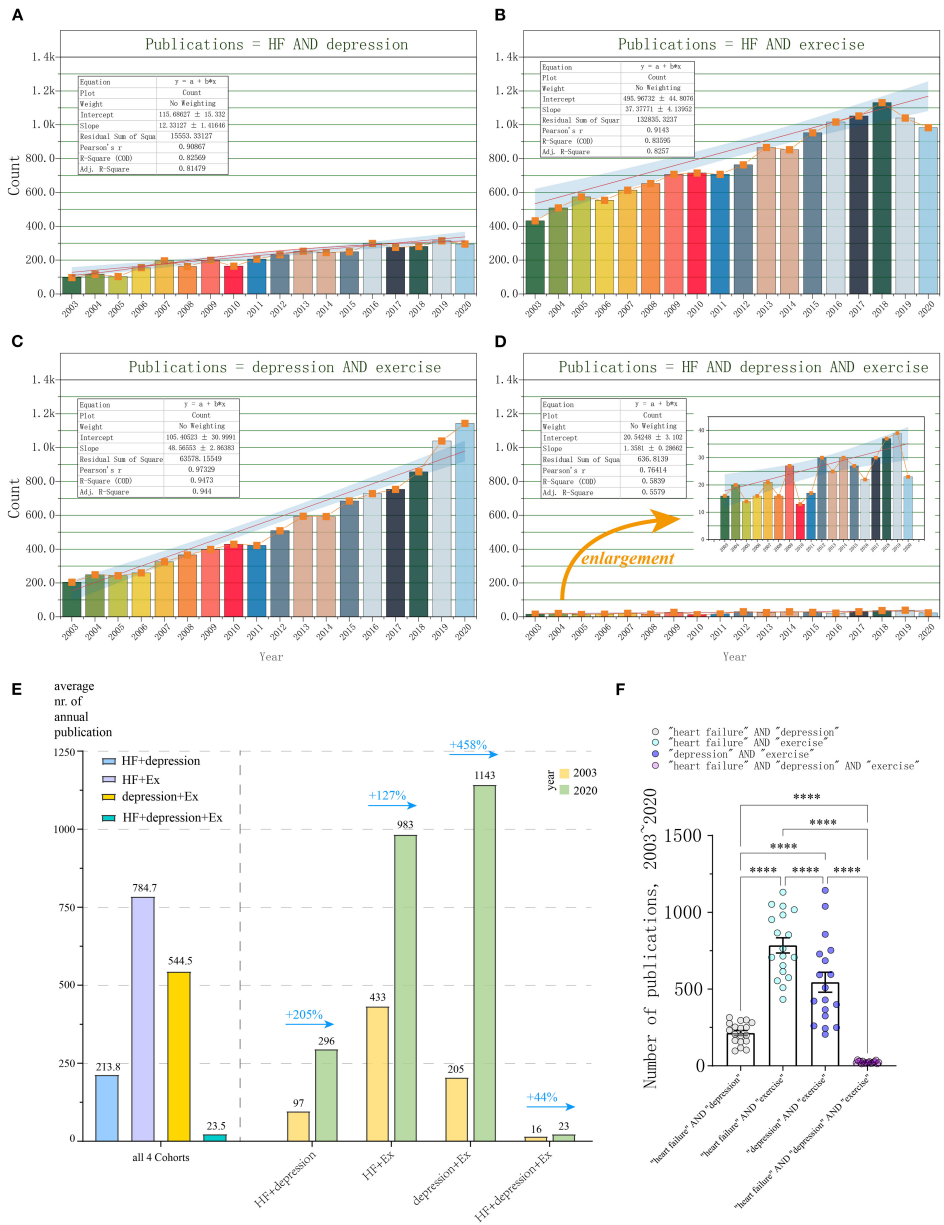
Characteristic analysis of relative research interest (publication number and trends) from 01/2003 to 12/2020. **(A)** Cohort 1: publications matched with “heart failure” AND “depression” (HF+depression); **(B)** Cohort 2: “heart failure” AND “exercise” (HF+Ex); **(C)** Cohort 3: “heart failure” AND “depression” AND “exercise” (depression+Ex); **(D)** Cohort 4: “heart failure” AND “depression” (HF+depression+Ex); **(E)** Annual output analysis of four Cohorts; **(F)** Statistical analysis on the publication number of four Cohorts. Results of two-way ANOVAs are ^****^p < 0.0001, *F* = 122.5, for different comparisons.

Linear regression model was used to create the time curve of the cumulative number of publications from which an overall overview could be drawn and future trends could be predicted. From January 2003 to December 2020, a total of 3,848 publications matched with “heart failure” AND “depression” (keyword-driven Cohort 1). The general trend of publications from 2003 to 2020 followed a linear trend more closely than an exponential curve (Figure 1A). Through performing linear fitting, we obtained Pearson's coefficient of determination of *r* = 0.90867 and *r*^2^ = 0.82569 (Intercept: 115.69 ± 15.33, Slope: 12.33 ± 1.42). The annual publication increased from 97 of year 2003 to 296 of year 2020. Most research was published in 2019 (315, 8.1861%). The average annual publication volume was 213.8 (Figure 1E).

Over the same period, the number of publications met the criteria “heart failure” AND “exercise” (Cohort 2) is 15,125. There also has been a steadily increasing trend of global publications annually. The number of publications increased from 433 (2003) to 983 (2020). Most research was published in 2018 (1,131, 8.007%). The average annual publication volume was 784.7. Pearson's coefficient of determination of *r* = 0.9143 and *r*^2^ = 0.83596 (Intercept: 495.97 ± 44.81, Slope: 37.38.33 ± 4.14) were obtained through performing linear fitting (Figures 1B,E).

Additionally, a total of 9,801 publications were found to meet the criteria “depression” AND “exercise” (Cohort 3) during the same period. The number of annual publications steadily increased from 205 (2003) to 1,143 (2020), and these were also the 2 years with the highest and lowest annual publication, respectively. The average annual publication volume was 544.5. Pearson's coefficient of determination of *r* was 0.97329 and *r*^2^ was 0.9473, respectively (Intercept: 105.41 ± 30.99, Slope: 48.57 ± 2.86, Figures 1C,E).

We next analyzed the publications based on the criteria for “heart failure” AND “depression” AND “exercise” (Cohort 4). In total, 423 publications met the above criteria. The number of publications increased from 16 (2003) to 23 (2020). Most research was published in 2019 (39, 9.220%). The average annual publication volume was 23.5. Pearson's coefficient of determination of *r* was 0.7641 and *r*^2^ was 0.5839, respectively (Intercept: 20.54 ± 3.10, Slope: 1.36 ± 0.029, Figures 1D,E).

We found that unlike from the annual publication volume ranking, from 2003 to 2020, the growth rate of the annual output from high to low is Cohort 3 (+458%), Cohort 1 (+205%), Cohort 2 (+127%), and Cohort 4 (only +44%), respectively. This indicated the difference of relative research interest (RRI) in certain fields (Figure 1E). Finally, we did statistical analysis on the publication number of four sets of search keywords, and noted that there were significant differences between any two groups (two-way ANOVAs, ^****^p < 0.0001, *F* = 122.5, Figure 1F).

### Analysis of the Contributions of Countries/Regions, Institutions and Funders to Related Keyword-Driven Research

**Figure 2:**
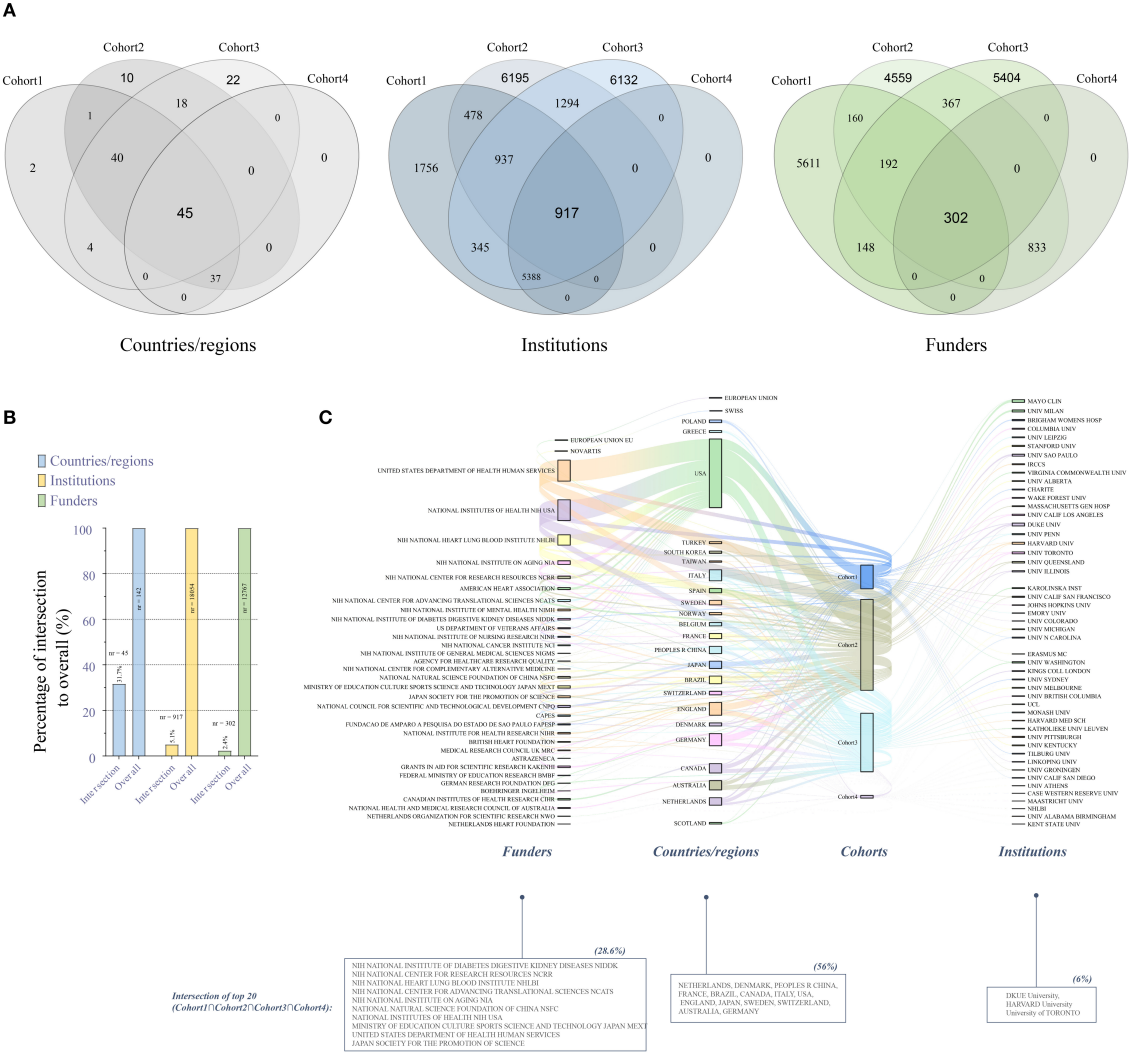
Contributions of countries/regions, institutions and funders to related keyword-driven research. **(A)** Venn diagram of the distributions of related keyword-driven research by countries/regions, institutions and funders from 2003 to 2020; **(B)** Percentage of intersection to overall keyword-driven research; **(C)** Sanky diagram of evaluation of the top 20 institutions and funders in the four keyword-driven cohorts.

Since the publications that met search criteria of the cohort 1 and cohort 4 (especially cohort 4) are obviously or extremely lower than the other two, in order to further explore the concerns on the concurrent HF-depression-exercise studies, we next assessed contributions of countries/regions, institutions and funders in related publications. Here, the Venn diagram (Figure 2A) showed the distributions/intersections (Cohort1nCohort2nCohort3nCohort4) of countries/regions, institutions and funders for the related keyword-driven research from 2003 to 2020, reflecting simultaneous concerns on related research fields in different dimensions. We noticed that about 31.7% of countries/regions have performed HF-depression-exercise research at the same time, but only 5.1 and 2.4% of institutions and funders participated in this perspective simultaneously (Figures 2A,B).

Additionally, we further evaluated the top 20 institutions and funders in the four cohorts (Figure 2C). We found that 56% countries/regions had conducted HF-depression-exercise research, and the United States remained the leading country in related keyword-driven research output. We also found that three institutions (DKUE University, HARVARD University and University of TORONTO) and 10 foundations (mainly from the United States) carried out research on HF-depression-exercise. In this cohort study, the proportions of countries/regions, institutions, and funders carrying out three studies at the same time are 56, 6, and 28.6%.

### Hotspot Analysis of Related Keyword-Driven Research Based on the Keyword Co-occurrence

**Figure 3:**
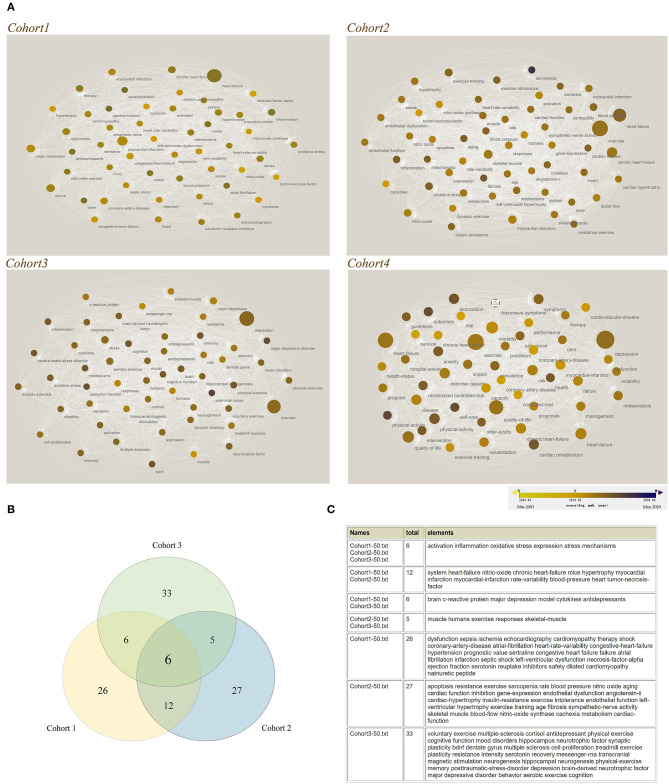
Hotspot analysis of related keyword-driven research based on the keyword co-occurrence. **(A)** Construct keywords sub-network for the top 50 keywords of four cohorts through VOSviewer and Cytoscape software. The diameter and the color of the dots reflected weighted co-occurrence and the changes in the timing, respectively; **(B)** Keyword intersection of three retrieval sequences; **(C)** Detailed information about the results of keyword intersection.

Visual knowledge map of keyword co-occurrence could reflect hot and frontier topics (25), therefore, we took the advantage of data visualization to analyze the research hotspots. First, we used VOSviewer and Cytoscape software to construct keywords sub-network for the top 50 keywords of four cohorts. The diameter and the color of the dots reflected the weighted co-occurrence and changes in the timeline, respectively (Figure 3A). Except the cohort 4, other three cohorts could provide important information about mechanism; therefore, we next performed the analysis of keyword co-occurrence in the first three cohorts in order to find more information about mechanism of the vicious circle (Figure 3A). Here, we noticed that the keyword intersection of three retrieval sequences contained 6 keywords (activation, inflammation, oxidative stress, expression, stress and mechanisms, Figure 3B). On the other hand, in fact, we cannot rule out the role of the intersection keywords of other retrieval sequences or the keywords of a single retrieval sequence since these research hotspots may be ignored but play critical roles in the other related research. We list all the results in the Figure 3C. The findings of the keyword intersection at least provide direct or important implications for the roles of inflammation and/or stress (including oxidative stress), which may be a common mechanism for exercise intervention to regulate the heart and central nervous system (CNS) through HBA.

### Research Hotspots and Frontiers of “Inflammation” and “Mitochondria” Studies Based on the Weighted Degree and Timeline

Given that mitochondrial dysfunction has been closely implicated in the development of HF and psychiatric disorders, or the mitochondria-induced protective effects of exercise (27–29), we reasoned that mitochondria would allow for novel in the development of exercise interventions for HF complicated with depression, and may be the potential target for pharmacological convention. Therefore, we next assessed the research hotspots and frontiers of studies associated with the mitochondria and the above findings.

**Figure 4:**
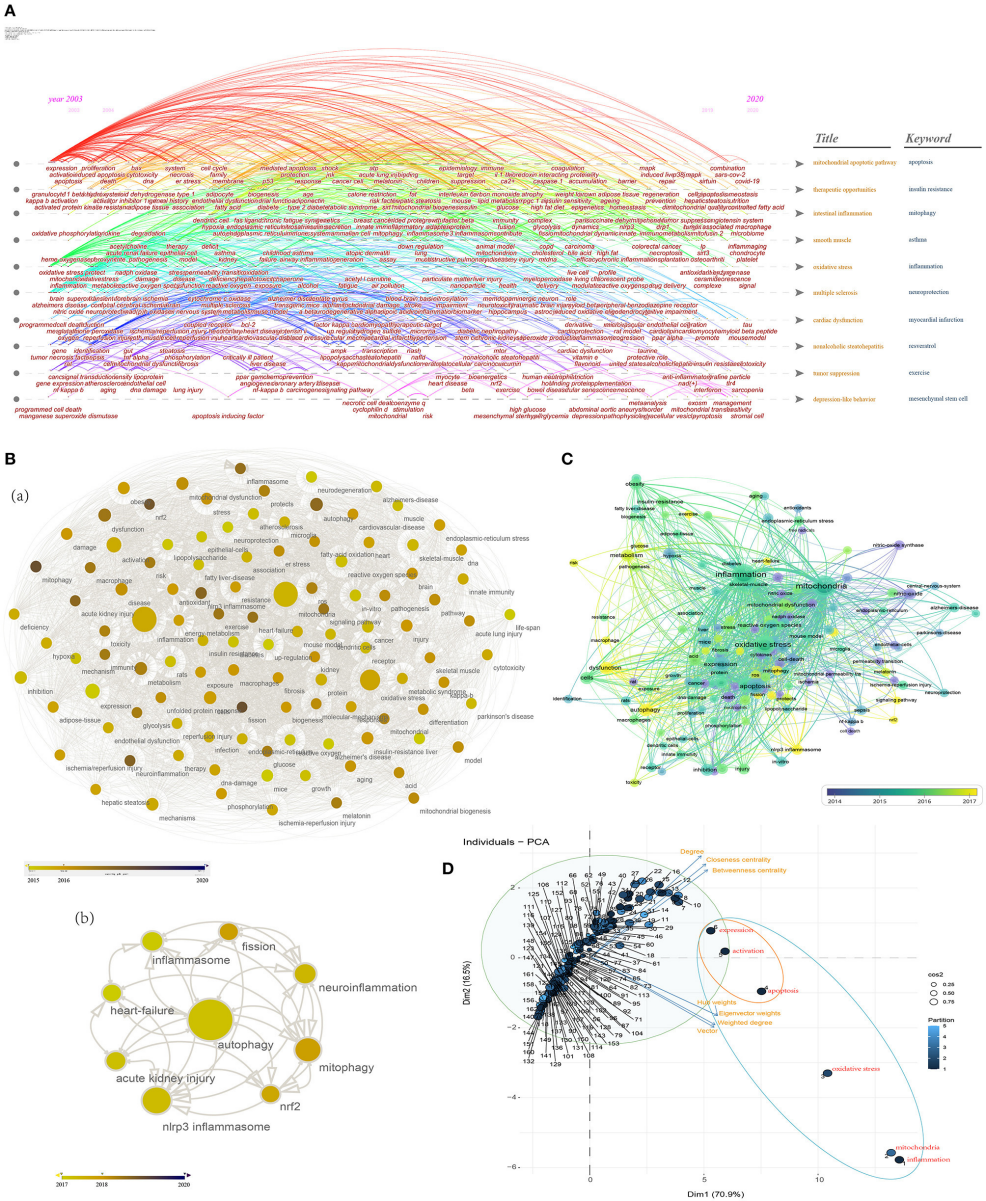
Research hotspots and frontiers of “inflammation” AND “mitochondria” studies. **(A)** Time-based overlay (sub-network and cluster map) visualization based on “Title & Keywords” (two topics) through using CiteSpace; **(B)** Analyze and visualize the research hotspots in recent years [2015 to 2020 (a), 2017 to 2020 (b)] through Cytoscape software; **(C)** Co-occurrence analysis through VOSviewer (the minimum number of occurrences of a keyword was over five); **(D)** PCA results of the analysis of the top 162 identified keywords provide highlights for the research programs. Keywords (all keywords) = Author keywords + Keywords Plus. The blue circle represents high eigenvector centrality, and the orange circle represents both high closeness centrality and high eigenvector centrality.

We first conducted time-based overlay visualization based on “Title & Keywords” (Figure 4A) through using CiteSpace, which can provide an interesting and revealing window to understand the evolution of research hotspots over time from the perspective of article titles and keywords. The “Title” topics included the following clusters: mitochondrial apoptotic pathway, therapeutic opportunities, intestinal inflammation, smooth muscle, oxidative stress, multiple sclerosis, cardiac dysfunction, non-alcoholic steatohepatitis, tumor suppression, and depression-like behavior. On the other hand, the “Keyword” topics included the following clusters: apoptosis, insulin resistance, mitophagy, asthma, inflammation, neuroprotection, myocardial infarction, resveratrol, exercise, and mesenchymal stem cell.

Table 1 shows network metrics for major co-occurrence keywords (top 50 based on the weighted degree) in the global network in the studies associated with “inflammation” and “mitochondria.” In addition, in terms of year, 39 published keywords were published in 2020 and are presented in Supplementary Table 1, such as covid-19, microbiome, signal, tau, amyloid beta peptide, antioxidant capacity, stromal cell, sarcopenia, sars-cov-2, blood-brain barrier (BBB), polyunsaturated fatty acid, and gene therapy, etc.

**Table 1:** Network metrics for top 50 co-occurrence keywords associated with inflammation & mitochondria.

	**Degree**	**Weighted degree**	**Closeness centrality**	**Normalized betweenness centrality**	**Eigenvector centrality**
Inflammation	161	11,957	1	0.00408	0.496312
Mitochondria	161	11,648	1	0.00408	0.483142
Oxidative stress	161	8,591	1	0.00408	0.385958
Apoptosis	161	5,507	1	0.00408	0.274758
Activation	160	4,134	0.99382716	0.00405	0.20389
Expression	161	3,439	1	0.00408	0.169877
nf-kappa-b	159	2,342	0.987730061	0.003959	0.116678
Dysfunction	161	2,205	1	0.00408	0.118276
Autophagy	159	2,191	0.987730061	0.003894	0.109738
Metabolism	159	1,928	0.987730061	0.003893	0.099918
Cells	147	1,791	0.92	0.003065	0.099045
Mechanisms	157	1,735	0.975757576	0.003763	0.090686
Inhibition	157	1,718	0.975757576	0.003698	0.09199
Cell-death	148	1,718	0.925287356	0.003189	0.086398
Mitochondrial dysfunction	158	1,640	0.981707317	0.003838	0.079141
Disease	153	1,503	0.952662722	0.003501	0.078879
Nitric-oxide	154	1,495	0.958333333	0.003596	0.074872
Obesity	133	1,471	0.851851852	0.002222	0.073671
Insulin-resistance	142	1,417	0.894444444	0.002696	0.068507
Reactive oxygen species	150	1,310	0.936046512	0.003236	0.067098
Injury	146	1,217	0.914772727	0.002931	0.063267
Mice	155	1,185	0.964071856	0.003629	0.059791
Cancer	145	1,072	0.90960452	0.002783	0.056977
Damage	143	1,039	0.899441341	0.002689	0.055777
Gene-expression	152	1,022	0.947058824	0.003427	0.049494
Stress	147	1,003	0.92	0.002967	0.050399
Protein	147	981	0.92	0.003211	0.046601
nlrp3 inflammasome	136	974	0.865591398	0.002505	0.04497
Death	135	967	0.860962567	0.002375	0.05062
Skeletal-muscle	134	932	0.856382979	0.002383	0.047803
Mitophagy	135	883	0.860962567	0.002489	0.043383
*In-vitro*	142	859	0.894444444	0.002776	0.046115
Pathway	131	847	0.842931937	0.002229	0.045964
*In-vivo*	145	837	0.90960452	0.003145	0.042539
Protects	133	818	0.851851852	0.002195	0.04351
Liver	133	816	0.851851852	0.002353	0.041454
Sepsis	121	801	0.800995025	0.001731	0.040394
Endoplasmic-reticulum stress	132	779	0.847368421	0.002306	0.037568
ROS	137	774	0.87027027	0.002547	0.040383
Macrophages	133	770	0.851851852	0.002316	0.037897
Nitric-oxide synthase	125	750	0.817258883	0.001858	0.034492
Antioxidant	133	744	0.851851852	0.00234	0.040985
Aging	133	744	0.851851852	0.002386	0.039744
Alzheimer's-disease	120	736	0.797029703	0.00177	0.037047
Brain	128	733	0.829896907	0.002221	0.037917
Atherosclerosis	129	682	0.834196891	0.002104	0.035664
Receptor	128	672	0.829896907	0.001978	0.034707
Model	127	659	0.825641026	0.001953	0.036709
Necrosis-factor-alpha	129	647	0.834196891	0.002242	0.033364
Neurodegeneration	116	638	0.781553398	0.001589	0.032902

In order to illustrate the frontier research hotspots, we next assessed co-occurrence keywords from 2015 to 2020 and from 2017 to 2020 through Cytoscape, respectively (Figure 4B). We found a large number of recent research hotspots linking to the mechanism for “inflammation” and “mitochondria,” e.g., fatty-acid oxidation, inflammasome, nlrp3 inflammasome, insulin resistance, endoplasmic-reticulum (er) stress, oxidative stress, energy-metabolism, metabolic syndrome, glycolysis, phosphorylation, epithelial-cells, endothelial dysfunction, mitochondrial dysfunction, autophagy, etc., especially some mechanisms directly related to mitochondria, such as mitochondrial biogenesis, mitophagy, fission, and reactive oxygen species (ROS) (Figure 4B(a)]. From 2017 to 2020, we further found 9 high-frequency keywords: autophagy, mitophagy, inflammasome, nlrp3 inflammasome, neuro inflammation, fission, nrf2, acute kidney injury, and heart-failure [Figure 4B(b)].

To investigate the weight of the research hotspots, we performed data visualization and PCA analysis. We did co-occurrence analysis through VOSviewer (the minimum number of occurrences of a keyword was over five), which calculates the total strength of co-occurrence links with other keywords, and selects the keywords with the greatest total strength. Figure 4C shows the weight of the top 162 identified keywords and their evolution over time. Ruling out the two keywords, inflammation and mitochondria, the top 15 keywords that appeared with the highest weight are oxidative stress, apoptosis, activation, expression, nf-kappa-b, dysfunction, autophagy, metabolism, cells, mechanisms, inhibition, cell-death, mitochondrial dysfunction, disease and nitric-oxide (Table 1). Finally, we performed PCA to explore the complex logic relationships between keywords (Figure 4D), so as to provide highlights for the research programs. Most of the points were arranged along the direction of closeness centrality and betweenness centrality, which reflect the difference of the degree of connection between points and provide clues for exploring the association mechanism. The corresponding keywords for the Arabic numerals in the Figure 4D can be found in the Supplementary Table 2. In the pseudo-2D space of PCA, we found that oxidative stress (except inflammation and mitochondria) has high weighted degree and eigenvector weights, suggesting that it has a key value in related research; and apoptosis (except activation and expression) has high weighted degree, eigenvector weights, closeness centrality, and betweenness centrality, indicating that it may play a chaining role in the related crosstalk mechanisms.

### Research Hotspots and Frontiers of “Oxidative Stress” and “Mitochondria” Studies Based on the Weighted Degree and Timeline

**Figure 5:**
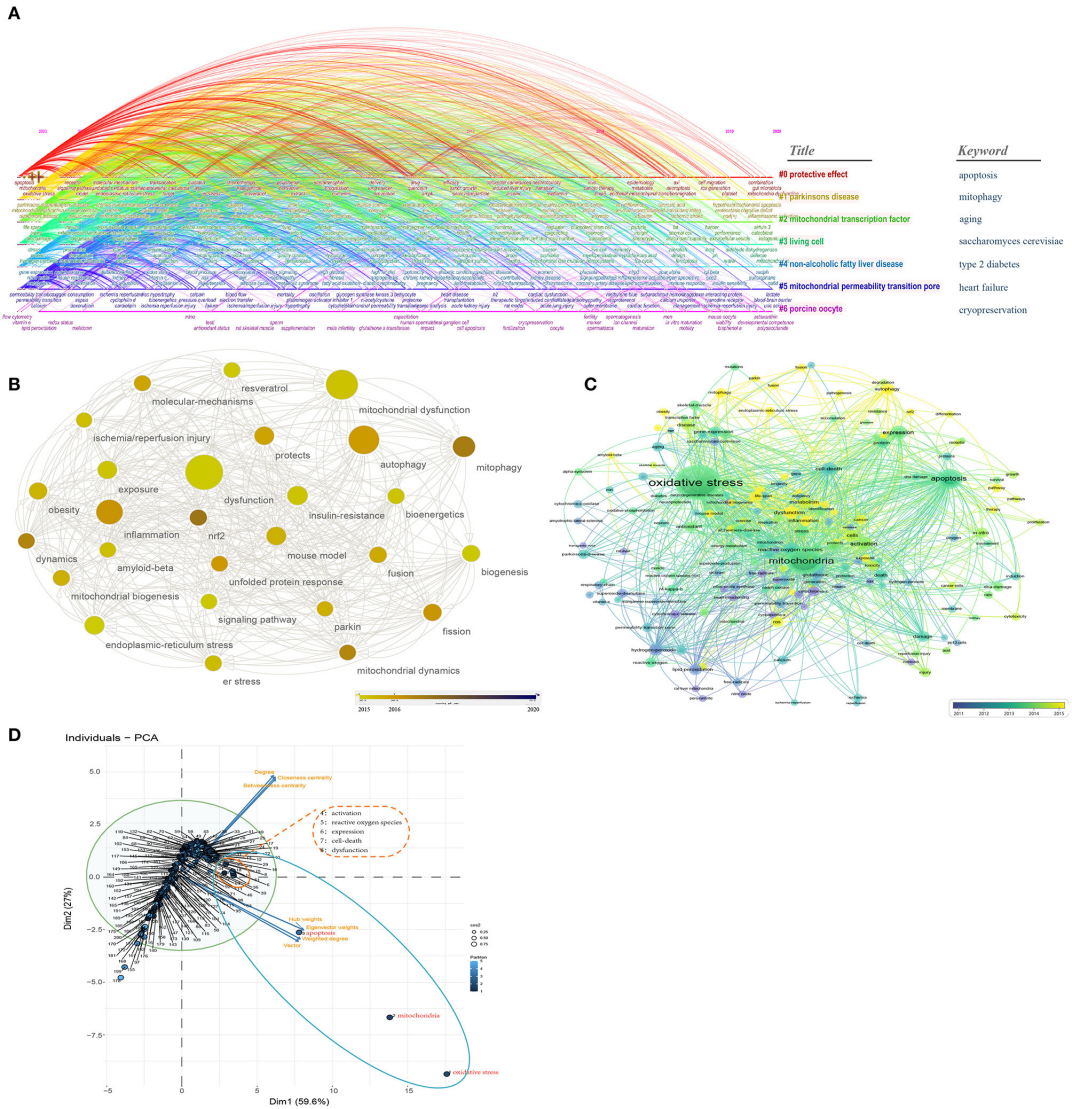
Research hotspots and frontiers of “oxidative stress” AND “mitochondria” studies. **(A)** Time-based overlay (sub-network and cluster map) visualization based on “Title & Keywords”; **(B)** Analyze and visualize the research hotspots in recent years (2015–2020); **(C)** Co-occurrence analysis through VOSviewer; **(D)** PCA results of the analysis of the top 200 identified keywords provide highlights for the research programs. The blue circle represents high eigenvector centrality, and the orange circle represents both high closeness centrality and high eigenvector centrality.

As shown in Figure 5A, the “Title” topics include the following clusters: protective effect, Parkinson's disease, mitochondrial transcription factor, living cell, non-alcoholic fatty liver disease, mitochondrial permeability transition pore, and porcine oocyte. In addition, using time-based overlay visualization based on paper keywords, the “Keyword” topics include: apoptosis, mitophagy, aging, saccharomyces cerevisiae, type 2 diabetes, heart failure, and cryopreservation. This is considered to be another evidence that mitochondria and oxidative stress are involved in heart failure and neurological disorders. Table 2 shows network metrics for major co-occurrence keywords (top 50 based on the weighted degree) in the global network in the studies associated with “oxidative stress” and “mitochondria.” Thirty-nine published keywords were published in 2020 and are presented in the Supplementary Table 3, such as cardiovascular disease, infarction, mitochondrial dynamics, sirtuin 3, mitochondrial transfer, mitochondrial apoptosis, gut microbiota, mitochondrial ro, mitochondrial calcium uniporter, BBB, mitochondrial homeostasis, astaxanthin, NASH, oligodendrocyte, long non-coding RNA, and d-galactose, etc.

**Table 2:** Network metrics for top 50 co-occurrence keywords associated with oxidative stress & mitochondria.

	**Degree**	**Weighted degree**	**Closeness centrality**	**Normalized betweenness centrality**	**Eigenvector centrality**
Oxidative stress	199	93,702	1	0.000393	0.574651
Mitochondria	199	67,196	1	0.000393	0.510334
Apoptosis	199	35,040	1	0.000393	0.310693
Activation	198	14,666	0.995	0.00039	0.138743
Reactive oxygen species	198	14,615	0.995	0.00039	0.136361
Expression	199	13,618	1	0.000393	0.130521
Cell-death	196	12,512	0.985149	0.000384	0.119335
Dysfunction	199	12,081	1	0.000393	0.111512
Mechanisms	198	9,372	0.995	0.00039	0.090042
Autophagy	199	9,058	1	0.000393	0.083795
Cells	194	9,019	0.97549	0.000371	0.087729
Metabolism	199	8,938	1	0.000393	0.08595
Mitochondrial dysfunction	199	8,851	1	0.000393	0.078987
Inhibition	197	8,544	0.99005	0.000374	0.079193
Damage	197	7,981	0.99005	0.000385	0.075774
Nitric-oxide	197	7,852	0.99005	0.000378	0.073
Hydrogen-peroxide	199	7,785	1	0.000393	0.074136
Death	196	7,750	0.985149	0.000381	0.075935
Lipid-peroxidation	199	7,605	1	0.000393	0.069439
ROS	199	7,580	1	0.000393	0.074157
Stress	198	7,165	0.995	0.000391	0.052955
Antioxidant	198	6,935	0.995	0.000389	0.067184
Glutathione	197	6,905	0.99005	0.000376	0.066193
*In-vitro*	198	6,878	0.995	0.000393	0.069043
Brain	199	6,685	1	0.000393	0.061112
Cytochrome-c	197	6,555	0.99005	0.000382	0.063404
Gene-expression	199	6,515	1	0.000393	0.064641
Disease	199	6,288	1	0.000393	0.060182
Protein	196	6,280	0.985149	0.000382	0.059258
Inflammation	197	6,185	0.99005	0.000374	0.063117
Alzheimer's-disease	197	5,992	0.99005	0.000375	0.055411
Aging	197	5,938	0.99005	0.000366	0.055224
Permeability transition	199	5,679	1	0.000393	0.052633
Parkinson's-disease	196	5,656	0.985149	0.000354	0.049544
Toxicity	193	5,502	0.970732	0.00034	0.052774
Skeletal-muscle	194	5,449	0.97549	0.000342	0.050652
Mice	198	5,348	0.995	0.000385	0.0494
Reactive oxygen	199	5,329	1	0.000393	0.049557
Injury	195	5,159	0.980296	0.000334	0.048301
Mitophagy	197	5,035	0.99005	0.000378	0.043454
*In-vivo*	198	5,024	0.995	0.000393	0.048667
Calcium	199	4,994	1	0.000393	0.048244
Permeability transition pore	199	4,891	1	0.000393	0.044824
DNA-damage	195	4,832	0.980296	0.000366	0.047154
Cancer	196	4,781	0.985149	0.000365	0.048848
Neurodegeneration	193	4,687	0.970732	0.000324	0.043704
Induced apoptosis	196	4,632	0.985149	0.000358	0.042516
Antioxidants	197	4,570	0.99005	0.000379	0.043345
Superoxide	198	4,493	0.995	0.000389	0.040948
Parkinson's disease	185	4,415	0.934272	0.000277	0.040018

In order to highlight the frontier research hotspots, we further assessed co-occurrence keywords from 2015 to 2020 through Cytoscape (Figure 5B). We found some recent research hotspots linking to the mechanism for “oxidative stress” and “mitochondria,” e.g., er stress, autophagy, mitochondrial dysfunction, mitochondrial biogenesis, mitophagy, fusion, fission, and parkin, etc. Figure 5C shows weight of the top 200 identified keywords and their evolution over time. Finally, we performed PCA to explore the logic relationships between keywords (Figure 5D). Same as the above PCA results, most of the points were arranged along the direction of closeness centrality and betweenness centrality. We found that, in the pseudo-2D space of PCA, apoptosis (except oxidative stress and mitochondria) has high weighted degree and eigenvector weights; and ROS, as well as cell death (except activation, expression and dysfunction) have high weighted degree, eigenvector weights, closeness centrality, and betweenness centrality, indicating that the logical relationship of mechanisms has changed. The corresponding key words for the Arabic numerals in the Figure 5D can be found in the Supplementary Table 4.

## Discussion

In clinic, depression is a common comorbidity of HF, which has already been proved to be associated with significant mortality and morbidity. About 40% of HF patients were affected by depression and with up to 75% of HF patients were reported being with elevated depressive symptoms (30, 31). Additionally, a previous etiological study has shown that the presence of depression doubles the risk of developing new CVD (32). Therefore, there is no doubt as to whether CVD causes more depression or whether depression causes more CVD and a worse prognosis for CVD (33). In fact, the prevalence of depression in cardiac patients has been noted for more than 50 years (34, 35). Consistent with above notion, we found that from January 2003 to December 2020, a total of 3,848 publications matched with “heart failure” AND “depression,” and the average annual publication volume was 213.8 (Figure 1). Fortunately, the annual publication volume increased year by year, indicating that the research is getting attention.

Compared with expensive pharmacological treatment with series side effects in cardiac disease patients (13, 33), physical activity and exercise have been recommended as a safe and effective therapy for the prevention and treatment of numerous diseases, including CVD and depression (12, 13, 36). These findings line with our study that exercise has been widely concerned in the study of heart failure and depression, respectively (Figure 1). Since exercise alone has been shown to be effective in intervening heart failure and depression, it would be of great significance to study the mechanism of exercise through blocking the vicious circle for the treatment of HF patients with depression. Therefore, finding the mechanism of simultaneous intervention of exercise on the heart and brain is an important way to achieve this goal. However, we found that the average annual publication volume of Cohort 4, from 2003 to 2020, is only 23.5 and unstable, with an annual output growth rate of +44%, but the linear regression coefficient *r* is 0.7641 (Figure 1), indicating that although much attention regarding to the HF-depression-exercise has been paid, the effort was far from enough. On the other hand, in the field of depression research, its growth rate is significantly higher (+458%) than that of CVD (+205%), with coefficient of determination of *r* reaching 0.97329, increasing from 205 annual publication in 2003 to 1,143 annual publication in 2020, which is close to the publication volume of CVD. Especially in 2019 and 2020, its annual publication keeps an increasing trend. These observations may be reflective of receiving more concern of exercise intervention for depression during the COVID-19 pandemic. Furthermore, we also found that the analysis of the distribution of countries/regions and institutions supported the view of the above publication volume analysis. We noticed that the proportion of leading institutions which performed simultaneous HF-depression-exercise research is still very low (6%). As described above, it is indeed urgent and necessary for HF-depression-exercise research.

To further investigate the common mechanism by which exercise may interfere with both the heart and brain, we conducted a detailed analysis of the publications on HF-depression, HF-exercise and depression-exercise in the WOS database from 2003 to 2020. Interestingly, a special attention has been paid by scientific community during a specific time-period and these keywords could represent the research hotspots and frontiers of a special field or subject in one period (37). Therefore, we analyzed the intersection of keywords in the field of HF and depression research related to exercise, with a view to find the possible common mechanisms of exercise therapy involved in HF with depression.

Six keywords were identified in the intersection of the analysis (activation, inflammation, oxidative stress, expression, stress and mechanisms, Figures 3B,C). In our hands, it is just as likely that these results at least have direct and important implications for the roles of inflammation and stress, particularly in light of oxidative stress, in the relationship between exercise, heart failure and depression; it also strongly suggested that these may be the common mechanism by which exercise intervention modulates the heart and CNS at both endpoints of the vicious circle. On the other hand, in fact, the intersection keywords of other retrieval sequence or the keywords of a single retrieval sequence could not be ruled out in the current observation, because these research hotspots may be ignored in one study, but play a key role in another (Figure 3C).

As we have demonstrated here that exercise as a treatment is indeed required for HF patients with depression, as well as the previous findings on the role of mitochondria in HF, psychiatric disorders, inflammation and oxidative stress (11, 27–29), the mechanism of how does inflammation or oxidative stress and activation of mitochondria coordinate has yet to be fully evaluated, we reasoned that mitochondria would allow for novel in the development of exercise interventions for HF complicated with depression, and may be the potential target for drug treatment and design.

The analysis of “mitochondria AND inflammation” through a variety of software provided some interesting and revealing windows for us to study the role of exercise and inflammation in the intervention of HF with depression through mitochondria, e.g., oxidative stress, apoptosis, er stress, endothelial dysfunction, and nitric-oxide, *etc*., especially some mechanisms directly related to mitochondria, such as mitochondrial biogenesis, mitophagy, fission, and ROS (Figure 4). Finally, we analyzed the significance of these research hotspots in related mechanisms through big data analysis (Figure 4D).

Furthermore, the analysis of “mitochondria AND oxidative stress” was also initiated (Figure 5). Our result is considered to be additional evidence that mitochondria and oxidative stress are involved in heart failure and neurological disorders, and simultaneously provide an overview and highlight the potential research hotspots, such as mPTP, sirtuin, apoptosis, mitochondrial calcium uniporter, mitochondrial homeostasis, mitophagy, fusion, fission, etc.

## Conclusion

We noted that inflammation, stress, oxidative stress, apoptosis, reactive oxygen species, cell death, and the mechanisms related to mitochondrial biogenesis/homeostasis, could be regarded as the primary mechanism targets to study the simultaneous intervention of exercise on the heart and brain of HF patients with depression.

It is needed to be mentioned that previous research linking HF-depression-exercise have been carried out, but the publication volume is very few. Importantly, the research areas are mainly concentrated in self-care, physical activity, outcomes, meta-analysis, etc. (14–16), and there is a lack of mechanism research and interdisciplinary cooperation. As a result, some important mechanisms in related fields cannot be applied in this study. In summary, exercise as an effective measure to intervene at both ends of the vicious cycle of HF patients with depression, should arouse more attention. In the current study, we demonstrated the possible primary mechanistic targets for exercise to simultaneously interfere with the heart and brain of HF patients with depression. We believe that the detection of mitochondria-related mechanisms with affecting inflammation and oxidative stress in response to exercise, could boost the attention of other researchers and clinicians, promote the development of therapeutic mechanisms of exercise in HF patients with depression, and open up new avenues for designing more novel potential drugs to block HBA vicious circle (especially for those HF patients who cannot perform exercise training).

## Data Availability Statement

The original contributions presented in the study are included in the article/Supplementary Material, further inquiries can be directed to the corresponding author/s.

## Author Contributions

YW designed the study, analyzed data, and wrote the manuscript. YJ and ML collected and analyzed data. SJ collected data. HZ designed the study, analyzed data, and wrote and edited the manuscript. All authors contributed to the article and approved the submitted version.

## Conflict of Interest

The authors declare that the research was conducted in the absence of any commercial or financial relationships that could be construed as a potential conflict of interest.
