# Supplementary material for: Hotspot and Frontier Analysis of Exercise Training Therapy for Heart Failure Complicated With Depression Based on Web of Science Database and Big Data Analysis
Source: Front Cardiovasc Med. 2021 May 19;8:665993. doi: 10.3389/fcvm.2021.665993 (PMC8169975; doi:10.3389/fcvm.2021.665993)
Supplement: Supplementary file 1 [file Table_1.pdf]

Supplemental Table 1. Co-occurrence analysis of "inflammation" AND "mitochondria" studies in 2020

Count	Centrality	year	Keywords
4	0	2020	antioxidant capacity
4	0	2020	diabetic retinopathy
4	0	2020	cyclic gmp amp
4	0	2020	dehydrogenase
4	0	2020	dna repair
4	0	2020	glucagon like peptide 1
4	0	2020	cardiotoxicity
4	0	2020	blood-brain barrier
4	0	2020	biology
4	0	2020	glucose homeostasis
4	0	2020	gene therapy
4	0	2020	increase
4	0	2020	combination
4	0	2020	disruption
5	0	2020	cystic fibrosis
5	0	2020	lipid droplet
5	0	2020	sars-cov-2
5	0	2020	inflammaging
5	0	2020	myocardial ischemia
5	0	2020	ultrafine particle
5	0	2020	management
5	0	2020	diabetic cardiomyopathy
5	0	2020	chondrocyte
5	0	2020	fluorescence
5	0	2020	platelet
5	0	2020	nutrition
6	0	2020	tau
6	0	2020	polyunsaturated fatty acid
6	0	2020	sensitivity
6	0	2020	amyloid beta peptide
6	0	2020	tlr4
6	0	2020	renin angiotensin system
6	0	2020	mousemodel
6	0	2020	tumor associated macrophage
7	0	2020	microbiome
8	0	2020	signal
8	0	2020	stromal cell
11	0	2020	sarcopenia
12	0	2020	covid-19
