# Supplementary material for: Hotspot and Frontier Analysis of Exercise Training Therapy for Heart Failure Complicated With Depression Based on Web of Science Database and Big Data Analysis
Source: Front Cardiovasc Med. 2021 May 19;8:665993. doi: 10.3389/fcvm.2021.665993 (PMC8169975; doi:10.3389/fcvm.2021.665993)
Supplement: Supplementary file 2 [file Table_2.pdf]

Label	ID	partiton	vector	AllDegree	WeightedAllDegree	AllclosenessCentrality	BetweennessCentrality	HubWeights	AuthorityWeights
inflammation	1	1	2363	161	11957	1	0.004079848	0.496312134	0.496312193
mitochondria	2	3	2335	161	11648	1	0.004079848	0.483142349	0.483142297
oxidative stress	3	2	1650	161	8591	1	0.004079848	0.385957841	0.385957836
apoptosis	4	1	1017	161	5507	1	0.004079848	0.274758001	0.274757997
activation	5	1	747	160	4134	0.99382716	0.004049813	0.203889633	0.203889632
expression	6	1	675	161	3439	1	0.004079848	0.169876515	0.169876513
nf-kappa-b	7	2	423	159	2342	0.987730061	0.003959244	0.116678008	0.116678006
dysfunction	8	4	396	161	2205	1	0.004079848	0.11827609	0.118276089
autophagy	9	1	387	159	2191	0.987730061	0.003893561	0.109738303	0.109738302
metabolism	10	3	365	159	1928	0.987730061	0.003893062	0.099918037	0.099918038
cells	11	4	364	147	1791	0.92	0.003064897	0.099044716	0.099044715
mechanisms	12	4	326	157	1735	0.975757576	0.003762927	0.090685706	0.090685706
inhibition	13	1	312	157	1718	0.975757576	0.003698468	0.091990244	0.091990243
cell-death	14	5	306	148	1718	0.925287356	0.003189401	0.086398435	0.086398436
mitochondrial dysfunction	15	2	313	158	1640	0.981707317	0.003837851	0.079141316	0.079141315
disease	16	4	298	153	1503	0.952662722	0.003500575	0.078879472	0.078879472
nitric-oxide	17	2	284	154	1495	0.958333333	0.003595648	0.074871516	0.074871517
obesity	18	3	261	133	1471	0.851851852	0.002221724	0.073671204	0.073671204
insulin-resistance	19	3	273	142	1417	0.894444444	0.002695585	0.068507289	0.068507289
reactive oxygen species	20	2	245	150	1310	0.936046512	0.003236151	0.067098039	0.06709804
injury	21	4	219	146	1217	0.914772727	0.002931373	0.063266569	0.063266569
mice	22	4	231	155	1185	0.964071856	0.003628536	0.059790619	0.059790619
cancer	23	1	214	145	1072	0.90960452	0.002782719	0.056977004	0.056977003
damage	24	4	193	143	1039	0.899441341	0.002689428	0.055776942	0.055776942
gene-expression	25	3	222	152	1022	0.947058824	0.003427402	0.049494189	0.049494189
stress	26	1	187	147	1003	0.92	0.002967459	0.050399367	0.050399366
protein	27	1	200	147	981	0.92	0.003210766	0.046600634	0.046600634
nlrp3 inflammasome	28	1	183	136	974	0.865591398	0.002504668	0.044970281	0.044970282
death	29	1	179	135	967	0.860962567	0.002375084	0.050620282	0.050620282
skeletal-muscle	30	3	182	134	932	0.856382979	0.002382784	0.047803434	0.047803435
mitophagy	31	1	145	135	883	0.860962567	0.002489017	0.043383428	0.043383427
in-vitro	32	1	184	142	859	0.894444444	0.00277636	0.046115	0.046115
pathway	33	1	152	131	847	0.842931937	0.002229131	0.045963559	0.045963558
in-vivo	34	2	173	145	837	0.90960452	0.003144606	0.042538789	0.042538789
protects	35	4	128	133	818	0.851851852	0.002195401	0.043509584	0.043509584
liver	36	4	148	133	816	0.851851852	0.002352928	0.041453994	0.041453994
sepsis	37	4	138	121	801	0.800995025	0.001731481	0.040393577	0.040393577
endoplasmic-reticulum stress	38	5	148	132	779	0.847368421	0.002305927	0.037567579	0.037567579
ros	39	1	138	137	774	0.87027027	0.002547326	0.040383251	0.040383251
macrophages	40	1	137	133	770	0.851851852	0.002316395	0.037897205	0.037897205
nitric-oxide synthase	41	2	147	125	750	0.817258883	0.00185814	0.034491621	0.034491621
antioxidant	42	2	130	133	744	0.851851852	0.002339943	0.040984665	0.040984665
aging	43	3	133	133	744	0.851851852	0.002386202	0.039744255	0.039744255
alzheimers-disease	44	2	135	120	736	0.797029703	0.001770161	0.037046719	0.037046719
brain	45	2	144	128	733	0.829896907	0.002220845	0.037917237	0.037917237
atherosclerosis	46	3	125	129	682	0.834196891	0.002103671	0.035663982	0.035663981
receptor	47	1	134	128	672	0.829896907	0.001978142	0.03470684	0.03470684
model	48	4	125	127	659	0.825641026	0.001953177	0.03670938	0.03670938
necrosis-factor-alpha	49	3	128	129	647	0.834196891	0.002242427	0.033363807	0.033363807
neurodegeneration	50	2	119	116	638	0.781553398	0.001588919	0.032902208	0.032902208
pathogenesis	51	4	116	133	622	0.851851852	0.002278004	0.032870061	0.032870061
activated protein-kinase	52	3	121	129	618	0.834196891	0.00213906	0.029024246	0.029024246
cytochrome-c	53	1	117	124	615	0.813131313	0.001868235	0.03217766	0.03217766
rats	54	4	111	125	606	0.817258883	0.001915784	0.033058626	0.033058626
tnf-alpha	55	5	111	133	604	0.851851852	0.002190468	0.028611541	0.028611541
adipose-tissue	56	3	115	99	587	0.721973094	8.99E-04	0.031129196	0.031129196
ischemia-reperfusion injury	57	5	112	114	583	0.774038462	0.001432372	0.031342837	0.031342837
mouse model	58	2	120	121	574	0.800995025	0.001882684	0.031795024	0.031795024
hypoxia	59	5	102	125	572	0.817258883	0.001997425	0.030042302	0.030042302
cytokines	60	1	104	124	571	0.813131313	0.001915251	0.031073544	0.031073544
heart	61	5	102	122	571	0.805	0.001750095	0.032400453	0.032400453
induced apoptosis	62	1	111	129	567	0.834196891	0.00214609	0.025435142	0.025435142
tumor-necrosis-factor	63	2	108	128	546	0.829896907	0.002187025	0.027441405	0.027441405
metabolic syndrome	64	3	93	111	540	0.763033175	0.001213845	0.026445043	0.026445043
induction	65	1	100	114	534	0.774038462	0.001435383	0.027893427	0.027893427
dna-damage	66	5	102	123	534	0.809045226	0.001709146	0.028639665	0.028639665
diabetes	67	3	86	117	526	0.785365854	0.001601466	0.026232679	0.026232679
lipopolysaccharide	68	4	85	121	523	0.800995025	0.001719267	0.026048579	0.026048578
proliferation	69	1	102	112	517	0.766666667	0.001441412	0.029789246	0.029789246
neuroinflammation	70	2	92	102	515	0.731818182	9.79E-04	0.025141991	0.025141992
microglia	71	2	86	104	514	0.73853211	0.001213665	0.026961916	0.026961915
reactive oxygen	72	2	100	122	512	0.805	0.001776767	0.02685448	0.026854481
glutathione	73	4	88	112	503	0.766666667	0.001473079	0.027295427	0.027295427
inflammasome	74	1	81	112	500	0.766666667	0.001287044	0.022928868	0.022928869
phosphorylation	75	1	90	130	492	0.838541667	0.002180688	0.02594008	0.02594008
kappa-b	76	4	86	134	490	0.856382979	0.002357769	0.024424472	0.024424473
antioxidants	77	2	86	117	485	0.785365854	0.001633534	0.024485766	0.024485766
nf-kappa b	78	1	74	120	478	0.797029703	0.001638622	0.025155966	0.025155965
insulin resistance	79	3	79	99	476	0.721973094	9.77E-04	0.022732789	0.022732789
er stress	80	5	83	117	470	0.785365854	0.001570475	0.022526811	0.02252681
nadph oxidase	81	2	81	107	464	0.748837209	0.001250376	0.023214469	0.023214469
melatonin	82	2	74	111	458	0.763033175	0.001364993	0.026449918	0.026449918
fibrosis	83	4	91	117	455	0.785365854	0.001435607	0.02434674	0.02434674
innate immunity	84	1	94	115	454	0.777777778	0.001604837	0.021062991	0.02106299
nitric oxide	85	2	78	107	452	0.748837209	0.001249916	0.023540448	0.023540449
mechanism	86	1	81	118	446	0.789215686	0.001662916	0.023597751	0.023597751
neuroprotection	87	2	81	105	446	0.741935484	0.001039308	0.024299504	0.024299504
alzheimers disease	88	2	81	95	441	0.709251101	8.70E-04	0.023671071	0.023671071
lipid-peroxidation	89	2	96	104	440	0.73853211	0.001261862	0.024492798	0.024492798
toxicity	90	4	81	93	435	0.703056769	8.15E-04	0.024582918	0.024582918
growth	91	1	83	107	434	0.748837209	0.001274243	0.022861944	0.022861943
release	92	1	77	104	428	0.73853211	0.00114401	0.021627736	0.021627736
mitochondrial biogenesis	93	3	70	118	412	0.789215686	0.001638768	0.018961982	0.018961981
differentiation	94	3	80	106	400	0.74537037	0.00118793	0.020862226	0.020862227
superoxide	95	2	71	106	394	0.74537037	0.001301748	0.021030839	0.021030839
endothelial dysfunction	96	2	69	103	392	0.735159817	0.001057991	0.018870674	0.018870675
exercise	97	3	69	93	391	0.703056769	8.58E-04	0.019906208	0.019906208
endothelial-cells	98	2	77	118	388	0.789215686	0.001750376	0.01892774	0.01892774
macrophage	99	1	72	110	387	0.759433962	0.001298514	0.018444961	0.018444961
pathways	100	1	66	104	387	0.73853211	0.00120135	0.018804362	0.018804362
exposure	101	4	72	92	384	0.7	7.92E-04	0.022346666	0.022346665
biogenesis	102	3	69	101	379	0.728506787	0.001134227	0.019244362	0.019244362
acute kidney injury	103	4	67	97	378	0.715555556	9.72E-04	0.020666587	0.020666588
dna	104	1	82	104	374	0.73853211	0.001114197	0.01977596	0.01977596
gene	105	1	79	110	371	0.759433962	0.00136229	0.0181819	0.018181901
up-regulation	106	2	74	108	370	0.752336449	0.001335717	0.017344771	0.017344771
calcium	107	5	70	105	367	0.741935484	0.00114321	0.019174426	0.019174426
parkinsons-disease	108	2	67	94	366	0.706140351	7.91E-04	0.017635931	0.017635931
responses	109	1	74	96	363	0.712389381	9.22E-04	0.019160242	0.019160242
necrosis	110	5	63	105	357	0.741935484	0.001254479	0.017694018	0.017694018
deficiency	111	4	69	104	356	0.73853211	0.001159933	0.01848475	0.01848475
molecular-mechanisms	112	5	67	115	356	0.777777778	0.001699355	0.017267136	0.017267136
therapy	113	1	72	109	351	0.755868545	0.001200879	0.017327431	0.017327431
acute lung injury	114	1	62	95	349	0.709251101	8.66E-04	0.019123223	0.019123222
survival	115	1	62	108	346	0.752336449	0.001443954	0.016848338	0.016848338
endoplasmic-reticulum	116	5	71	102	346	0.731818182	0.00112859	0.017066621	0.017066621
hydrogen-peroxide	117	2	72	101	342	0.728506787	0.001056685	0.018266834	0.018266834
septic shock	118	4	59	88	342	0.688034188	7.00E-04	0.015754992	0.015754992
cytotoxicity	119	4	63	97	339	0.715555556	8.75E-04	0.018378731	0.018378731
glucose	120	3	60	101	335	0.728506787	0.001048432	0.016936536	0.016936536
ischemia/reperfusion injury	121	5	58	95	335	0.709251101	8.45E-04	0.018031115	0.018031116

kidney	122	4	57	94	333	0.706140351	7.63E-04	0.017642108	0.017642108
ıitochondrial permeability transitic	123	5	66	94	331	0.706140351	9.42E-04	0.016812739	0.016812739
signaling pathway	124	5	55	109	331	0.755868545	0.001337968	0.017899017	0.017899017
rat	125	4	59	103	330	0.735159817	0.001283379	0.016320193	0.016320193
epithelial-cells	126	1	72	108	328	0.752336449	0.001286666	0.017897918	0.017897917
free radicals	127	2	50	110	327	0.759433962	0.001436418	0.015029677	0.015029677
association	128	3	70	98	323	0.71875	0.001016395	0.017781991	0.017781991
reperfusion injury	129	5	62	91	321	0.696969697	7.65E-04	0.016268004	0.016268004
cardiovascular-disease	130	3	64	91	318	0.696969697	9.25E-04	0.017085819	0.017085818
infection	131	1	69	92	316	0.7	7.62E-04	0.016451326	0.016451326
ischemia	132	5	62	87	314	0.685106383	8.12E-04	0.017283406	0.017283406
identification	133	1	73	90	313	0.693965517	8.04E-04	0.018570066	0.018570066
heart-failure	134	5	56	100	312	0.725225225	8.83E-04	0.016915896	0.016915896
permeability transition pore	135	5	58	106	311	0.74537037	0.001543423	0.015256616	0.015256617
muscle	136	3	58	92	310	0.7	7.03E-04	0.016556938	0.016556938
risk	137	3	70	91	308	0.696969697	7.58E-04	0.016341791	0.016341791
skeletal muscle	138	3	53	88	307	0.688034188	8.07E-04	0.015647175	0.015647176
acid	139	4	65	98	307	0.71875	9.73E-04	0.016364597	0.016364597
fatty liver disease	140	3	60	79	306	0.66255144	5.02E-04	0.015584074	0.015584074
fission	141	1	56	85	303	0.679324895	6.91E-04	0.015974873	0.015974873
glycolysis	142	1	51	89	302	0.690987124	8.38E-04	0.016558495	0.016558495
nrf2	143	2	52	96	299	0.712389381	9.55E-04	0.016287563	0.016287563
parkinsons disease	144	2	54	76	299	0.654471545	4.84E-04	0.014435688	0.014435688
cell death	145	5	51	102	299	0.731818182	0.0011838	0.015227629	0.015227629
permeability transition	146	5	62	97	299	0.715555556	9.19E-04	0.014944349	0.014944349
fatty-acid oxidation	147	3	58	93	296	0.703056769	8.79E-04	0.013659645	0.013659645
life-span	148	3	59	97	296	0.715555556	9.17E-04	0.014242647	0.014242647
smooth-muscle-cells	149	2	59	85	295	0.679324895	7.19E-04	0.014792442	0.014792442
resistance	150	1	58	94	292	0.706140351	8.63E-04	0.013993737	0.013993737
central-nervous-system	151	2	62	74	291	0.649193548	4.64E-04	0.014449328	0.014449328
mitochondrial	152	1	51	109	290	0.755868545	0.001601742	0.014599234	0.014599234
oxidative damage	153	2	57	96	286	0.712389381	0.001010459	0.013864255	0.013864256
proteins	154	1	58	100	282	0.725225225	0.001100344	0.014831735	0.014831735
signaling pathways	155	3	50	101	277	0.728506787	0.001143501	0.013490852	0.013490852
unfolded protein response	156	5	58	82	270	0.670833333	5.73E-04	0.013441646	0.013441646
immunity	157	1	51	94	264	0.706140351	9.47E-04	0.013008496	0.013008495
hepatic steatosis	158	3	50	84	253	0.676470588	6.82E-04	0.011367239	0.011367239
cytochrome-c release	159	5	51	85	252	0.679324895	7.85E-04	0.013363108	0.013363109
neutrophils	160	1	50	81	247	0.668049793	5.92E-04	0.013908542	0.013908542
energy-metabolism	161	3	53	91	245	0.696969697	9.23E-04	0.011761585	0.011761586
dendritic cells	162	1	53	79	223	0.66255144	5.27E-04	0.011056947	0.011056947
