# Supplementary material for: Hotspot and Frontier Analysis of Exercise Training Therapy for Heart Failure Complicated With Depression Based on Web of Science Database and Big Data Analysis
Source: Front Cardiovasc Med. 2021 May 19;8:665993. doi: 10.3389/fcvm.2021.665993 (PMC8169975; doi:10.3389/fcvm.2021.665993)
Supplement: Supplementary file 3 [file Table_3.pdf]

Supplemental Table 3. Co-occurrence analysis of "oxidative stress" AND "mitochondria" studies in 2020

Count	Degree	Centrality	Year	Keyword
**6**	11	0	2020	mcu
**6**	4	0	2020	cardiovasculardisease
**6**	7	0	2020	inflammatory bowel disease
**6**	11	0	2020	chain fatty acid
**6**	7	0	2020	high resolution respirometry
**6**	8	0	2020	anxiety
**6**	8	0	2020	lipid droplet
**6**	2	0	2020	apigenin
**6**	8	0	2020	long noncoding rna
**6**	6	0	2020	acute myeloid leukemia
**6**	7	0	2020	aldehyde dehydrogenase
**6**	5	0	2020	competence
**6**	12	0	2020	d-galactose
**6**	10	0	2020	mitochondrial calcium uniporter
**7**	10	0	2020	nash
**7**	7	0	2020	oligodendrocyte
**7**	10	0	2020	defense
**7**	15	0	2020	nonalcoholic fatty liver disease
**7**	48	0	2020	infarction
**7**	8	0	2020	semen quality
**7**	16	0	2020	mitochondrial transfer
**7**	7	0	2020	mitochondrial apoptosis
**7**	8	0	2020	lactate
**7**	9	0	2020	mitochondrial homeostasis
**7**	8	0	2020	pressure
**7**	8	0	2020	inflammaging
**7**	7	0	2020	blood-brain barrier
**7**	15	0	2020	cognitive deficit
**7**	3	0	2020	uric acid
**7**	9	0	2020	sirtuin 3
**7**	5	0	2020	osteoblast
**8**	6	0	2020	ketogenic diet
**8**	15	0	2020	parameter
**8**	7	0	2020	astaxanthin
**8**	5	0	2020	developmental competence
**8**	9	0	2020	mitochondrialdynamics
**9**	2	0	2020	polysaccharide
**9**	4	0	2020	nadph
**9**	9	0	2020	combination

**9**	3	0	2020	mitochondrial ro
**9**	3	0	2020	sulforaphane
**10**	6	0	2020	inflammasome activation
**11**	4	0	2020	gut microbiota
**12**	12	0	2020	nafld
**13**	2	0	2020	mitochondria dysfunction
