# Supplementary material for: Hotspot and Frontier Analysis of Exercise Training Therapy for Heart Failure Complicated With Depression Based on Web of Science Database and Big Data Analysis
Source: Front Cardiovasc Med. 2021 May 19;8:665993. doi: 10.3389/fcvm.2021.665993 (PMC8169975; doi:10.3389/fcvm.2021.665993)
Supplement: Supplementary file 4 [file Table_4.pdf]

Label	ID	partiton	vector	AllDegree	WeightedAllDegree	AllclosenessCentrality	BetweennessCentrality	HubWeights	AuthorityWeights
oxidative stress	1	2	18912	199	93702	1	3.93E-04	0.574650277	0.574650536
mitochondria	2	2	12449	199	67196	1	3.93E-04	0.510333977	0.51033377
apoptosis	3	3	5854	199	35040	1	3.93E-04	0.310693238	0.310693198
activation	4	3	2449	198	14666	0.995	3.90E-04	0.13874272	0.138742709
reactive oxygen species	5	1	2418	198	14615	0.995	3.90E-04	0.136361347	0.136361341
expression	6	3	2522	199	13618	1	3.93E-04	0.130520524	0.130520511
cell-death	7	1	2109	196	12512	0.985148515	3.84E-04	0.119335423	0.119335409
dysfunction	8	2	2069	199	12081	1	3.93E-04	0.111511626	0.111511613
mechanisms	9	3	1571	198	9372	0.995	3.90E-04	0.09004243	0.090042424
autophagy	10	2	1471	199	9058	1	3.93E-04	0.083794941	0.083794933
cells	11	5	1627	194	9019	0.975490196	3.71E-04	0.087729358	0.087729349
metabolism	12	2	1672	199	8938	1	3.93E-04	0.085950187	0.085950181
mitochondrial dysfunction	13	4	1576	199	8851	1	3.93E-04	0.078987025	0.078987007
inhibition	14	3	1419	197	8544	0.990049751	3.74E-04	0.079193343	0.079193338
damage	15	5	1358	197	7981	0.990049751	3.85E-04	0.075774338	0.075774331
nitric-oxide	16	1	1395	197	7852	0.990049751	3.78E-04	0.073000174	0.073000165
hydrogen-peroxide	17	1	1436	199	7785	1	3.93E-04	0.0741361	0.07413609
death	18	3	1242	196	7750	0.985148515	3.81E-04	0.075935437	0.075935431
lipid-peroxidation	19	5	1376	199	7605	1	3.93E-04	0.069438841	0.069438832
ros	20	3	1265	199	7580	1	3.93E-04	0.074157229	0.074157226
stress	21	2	1320	198	7165	0.995	3.91E-04	0.052954712	0.052954721
antioxidant	22	5	1162	198	6935	0.995	3.89E-04	0.067184267	0.067184261
glutathione	23	5	1184	197	6905	0.990049751	3.76E-04	0.066193267	0.066193262
in-vitro	24	3	1288	198	6878	0.995	3.93E-04	0.069043167	0.069043157
brain	25	4	1075	199	6685	1	3.93E-04	0.061112373	0.061112369
cytochrome-c	26	1	1061	197	6555	0.990049751	3.82E-04	0.063404192	0.063404187
gene-expression	27	2	1351	199	6515	1	3.93E-04	0.064640768	0.064640758
disease	28	2	1152	199	6288	1	3.93E-04	0.06018152	0.060181514
protein	29	2	1140	196	6280	0.985148515	3.82E-04	0.059257559	0.059257554
inflammation	30	3	1126	197	6185	0.990049751	3.74E-04	0.063116954	0.063116948
alzheimers-disease	31	4	1002	197	5992	0.990049751	3.75E-04	0.055411172	0.055411164
aging	32	2	956	197	5938	0.990049751	3.66E-04	0.05522416	0.055224156
permeability transition	33	1	942	199	5679	1	3.93E-04	0.052632959	0.052632954
parkinsons-disease	34	4	898	196	5656	0.985148515	3.54E-04	0.049543776	0.04954377
toxicity	35	5	954	193	5502	0.970731707	3.40E-04	0.052773731	0.052773724
skeletal-muscle	36	2	1012	194	5449	0.975490196	3.42E-04	0.050652475	0.050652469
mice	37	2	925	198	5348	0.995	3.85E-04	0.049400411	0.049400407
reactive oxygen	38	1	948	199	5329	1	3.93E-04	0.049556738	0.049556731
injury	39	5	863	195	5159	0.980295567	3.34E-04	0.048300758	0.048300754
mitophagy	40	2	775	197	5035	0.990049751	3.78E-04	0.043454107	0.043454101
in-vivo	41	4	958	198	5024	0.995	3.93E-04	0.048666958	0.048666951
calcium	42	1	814	199	4994	1	3.93E-04	0.048244484	0.048244481
permeability transition pore	43	1	820	199	4891	1	3.93E-04	0.044824265	0.04482426
dna-damage	44	3	861	195	4832	0.980295567	3.66E-04	0.047154163	0.047154157
cancer	45	3	862	196	4781	0.985148515	3.65E-04	0.04884801	0.048848007
neurodegeneration	46	4	731	193	4687	0.970731707	3.24E-04	0.043703711	0.043703707
induced apoptosis	47	3	787	196	4632	0.985148515	3.58E-04	0.042515521	0.042515516
antioxidants	48	5	774	197	4570	0.990049751	3.79E-04	0.04334538	0.043345376
superoxide	49	1	738	198	4493	0.995	3.89E-04	0.040948377	0.040948375
parkinsons disease	50	4	665	185	4415	0.9342723	2.77E-04	0.040017832	0.040017828
nf-kappa-b	51	3	806	195	4410	0.980295567	3.59E-04	0.043467983	0.043467977
superoxide-dismutase	52	5	808	196	4322	0.985148515	3.77E-04	0.040788438	0.040788433
complex-i	53	1	705	197	4277	0.990049751	3.65E-04	0.03756624	0.037566236
pathway	54	3	693	198	4200	0.995	3.83E-04	0.039001001	0.039000998
liver	55	5	701	191	4146	0.961352657	3.04E-04	0.038997174	0.038997171
life-span	56	2	722	195	4141	0.980295567	3.67E-04	0.03701881	0.037018806
heart	57	1	672	188	4039	0.947619048	2.94E-04	0.03729346	0.037293458
free radicals	58	1	601	198	3978	0.995	3.90E-04	0.034496078	0.034496076
phosphorylation	59	2	656	198	3898	0.995	3.88E-04	0.035668249	0.035668246
alzheimers disease	60	4	594	188	3746	0.947619048	3.03E-04	0.034946759	0.034946755
cytochrome-c release	61	1	626	193	3740	0.970731707	3.63E-04	0.035851875	0.035851871
mechanism	62	5	629	197	3620	0.990049751	3.86E-04	0.033007421	0.033007418
induction	63	3	566	190	3521	0.956730769	3.18E-04	0.034194064	0.034194061
generation	64	1	566	193	3507	0.970731707	3.24E-04	0.030809027	0.030809025
protects	65	5	540	189	3465	0.95215311	3.20E-04	0.032236118	0.032236116
nitric-oxide synthase	66	1	628	186	3346	0.938679245	2.84E-04	0.031403909	0.031403905
neuroprotection	67	4	517	191	3232	0.961352657	3.32E-04	0.031149669	0.031149667
mitochondrial permeability transitior	68	1	538	194	3214	0.975490196	3.60E-04	0.030065838	0.030065834
oxidative damage	69	4	581	195	3186	0.980295567	3.70E-04	0.023721925	0.023721927
free-radicals	70	1	533	197	3162	0.990049751	3.75E-04	0.028779503	0.0287795
ischemia	71	1	487	192	3090	0.966019417	3.12E-04	0.028355135	0.028355133
mouse model	72	4	545	193	3053	0.970731707	3.36E-04	0.029280074	0.02928007
endoplasmic-reticulum stress	73	3	551	193	3052	0.970731707	3.61E-04	0.028891396	0.028891393
insulin-resistance	74	2	607	187	3004	0.943127962	2.89E-04	0.029277663	0.029277659
respiratory-chain	75	1	514	195	2975	0.980295567	3.73E-04	0.026693085	0.026693081
rat	76	5	492	185	2970	0.9342723	2.86E-04	0.028022358	0.028022355
exposure	77	5	515	185	2918	0.9342723	2.73E-04	0.029625747	0.029625743
rats	78	5	523	189	2904	0.95215311	3.06E-04	0.028746482	0.028746478
cytotoxicity	79	3	505	180	2873	0.912844037	2.49E-04	0.030013856	0.030013852
oxygen	80	1	496	194	2863	0.975490196	3.33E-04	0.026418634	0.026418632
model	81	4	498	195	2835	0.980295567	3.72E-04	0.027450683	0.027450681
gene	82	2	572	196	2817	0.985148515	3.69E-04	0.027626778	0.027626775
growth	83	3	526	185	2734	0.9342723	2.74E-04	0.027139484	0.027139481
induced oxidative stress	84	5	502	195	2728	0.980295567	3.64E-04	0.020111171	0.020111174
fission	85	2	426	191	2706	0.961352657	3.38E-04	0.022630369	0.022630366
proteins	86	2	486	196	2698	0.985148515	3.81E-04	0.025945828	0.025945826
cytochrome-c-oxidase	87	4	504	191	2668	0.961352657	3.51E-04	0.024608733	0.02460873
neurodegenerative diseases	88	4	432	182	2655	0.921296296	2.66E-04	0.024336875	0.024336871
neurons	89	4	394	185	2648	0.9342723	2.79E-04	0.024596241	0.024596239
hypoxia	90	1	462	195	2621	0.980295567	3.45E-04	0.024256385	0.024256384
neurotoxicity	91	4	402	181	2592	0.917050691	2.77E-04	0.023331362	0.02333136
endoplasmic-reticulum	92	2	502	190	2567	0.956730769	3.42E-04	0.026483012	0.026483008
melatonin	93	5	371	190	2506	0.956730769	3.34E-04	0.023369116	0.023369115
ischemia-reperfusion injury	94	1	449	185	2489	0.9342723	2.93E-04	0.02332407	0.023324068
nadph oxidase	95	1	433	187	2472	0.943127962	3.14E-04	0.023699812	0.02369981
respiration	96	1	416	195	2445	0.980295567	3.62E-04	0.021921807	0.021921805
alpha-synuclein	97	4	402	163	2415	0.846808511	1.80E-04	0.021368879	0.021368876
activated protein-kinase	98	2	448	189	2388	0.95215311	3.51E-04	0.021936652	0.02193665
energy-metabolism	99	2	462	192	2354	0.966019417	3.57E-04	0.02285526	0.022855258
identification	100	2	489	191	2348	0.961352657	3.40E-04	0.023105352	0.023105351
cancer-cells	101	3	431	185	2314	0.9342723	2.85E-04	0.023678091	0.023678089
nitric oxide	102	1	362	192	2284	0.966019417	3.39E-04	0.020708461	0.02070846
biogenesis	103	2	387	189	2280	0.95215311	3.05E-04	0.019288727	0.019288724
fusion	104	2	349	184	2250	0.929906542	3.23E-04	0.018793826	0.018793824
saccharomyces-cerevisiae	105	2	497	174	2240	0.888392857	2.54E-04	0.021564769	0.021564766
hydrogen peroxide	106	1	356	192	2224	0.966019417	3.16E-04	0.020965019	0.020965018

dna	107	2	410	190	2217	0.956730769	3.21E-04	0.020662443	0.020662441
membrane	108	1	382	192	2214	0.966019417	3.34E-04	0.020528927	0.020528926
mutations	109	2	438	179	2194	0.908675799	2.67E-04	0.020124676	0.020124673
cell death	110	1	343	194	2177	0.975490196	3.62E-04	0.020542819	0.020542818
p53	111	3	342	187	2162	0.943127962	3.25E-04	0.02087037	0.020870368
mitochondrial	112	2	378	192	2161	0.966019417	3.29E-04	0.019447339	0.019447335
bcl-2	113	3	310	184	2148	0.929906542	2.85E-04	0.02049506	0.02049506
release	114	1	348	186	2146	0.938679245	3.17E-04	0.020232954	0.020232953
proliferation	115	3	376	183	2136	0.925581395	2.58E-04	0.021235903	0.021235901
obesity	116	2	413	171	2121	0.876651982	2.13E-04	0.020941868	0.020941866
involvement	117	3	332	191	2115	0.961352657	3.42E-04	0.020354385	0.020354383
diabetes	118	2	354	188	2077	0.947619048	2.96E-04	0.020108546	0.020108545
overexpression	119	2	346	187	2044	0.943127962	2.88E-04	0.019119852	0.01911985
lipid peroxidation	120	5	339	187	2042	0.943127962	3.13E-04	0.017591013	0.017591012
acid	121	5	363	181	2017	0.917050691	2.47E-04	0.019887335	0.019887334
nrf2	122	3	331	186	2003	0.938679245	2.95E-04	0.019176268	0.019176266
electron-transport	123	1	395	187	1967	0.943127962	3.17E-04	0.017799719	0.017799717
pathogenesis	124	4	341	182	1959	0.921296296	2.65E-04	0.018448132	0.018448131
exercise	125	2	342	175	1952	0.892376682	2.17E-04	0.017348159	0.017348157
necrosis	126	3	286	182	1949	0.921296296	2.81E-04	0.018833407	0.018833406
survival	127	3	325	187	1937	0.943127962	3.00E-04	0.018771916	0.018771915
pathways	128	3	298	184	1866	0.929906542	2.99E-04	0.018719406	0.018719405
dna damage	129	3	324	188	1854	0.947619048	3.16E-04	0.018290637	0.018290635
bax	130	3	272	178	1846	0.904545455	2.56E-04	0.017340995	0.017340994
brain mitochondria	131	1	299	177	1845	0.900452489	2.61E-04	0.014779929	0.014779926
bioenergetics	132	2	304	195	1843	0.980295567	3.61E-04	0.017427588	0.017427587
endothelial-cells	133	1	334	191	1843	0.961352657	3.43E-04	0.017872634	0.017872632
mitochondrial dynamics	134	2	283	181	1837	0.917050691	3.15E-04	0.014741844	0.014741841
er stress	135	3	302	183	1824	0.925581395	2.98E-04	0.017123169	0.017123167
up-regulation	136	3	319	193	1822	0.970731707	3.54E-04	0.01652789	0.016527887
senescence	137	2	322	181	1821	0.917050691	2.53E-04	0.018435151	0.01843515
mitochondrial biogenesis	138	2	312	185	1820	0.9342723	3.18E-04	0.015287016	0.015287013
manganese superoxide-dismutase	139	1	337	183	1817	0.925581395	2.78E-04	0.017209346	0.017209344
pc12 cells	140	4	263	175	1797	0.892376682	2.37E-04	0.016941026	0.016941024
superoxide-production	141	1	324	184	1794	0.929906542	3.02E-04	0.016545352	0.01654535
degradation	142	2	323	182	1793	0.921296296	2.92E-04	0.01591938	0.015919378
reperfusion injury	143	1	317	177	1793	0.900452489	2.30E-04	0.016578063	0.016578061
resistance	144	2	339	177	1765	0.900452489	2.51E-04	0.016775008	0.016775007
oxidative phosphorylation	145	1	313	191	1764	0.961352657	3.30E-04	0.015142493	0.015142492
peroxynitrite	146	1	288	178	1750	0.904545455	2.58E-04	0.015454887	0.015454887
electron-transport chain	147	1	300	177	1740	0.900452489	2.52E-04	0.014890879	0.014890877
caloric restriction	148	2	284	172	1736	0.880530973	2.31E-04	0.014529969	0.014529968
antioxidant enzymes	149	5	312	189	1732	0.95215311	3.01E-04	0.01569151	0.015691508
heart-failure	150	1	335	173	1727	0.884444444	2.10E-04	0.017764589	0.017764587
mitochondrion	151	1	318	193	1727	0.970731707	3.53E-04	0.013299746	0.013299742
dynamics	152	2	283	183	1719	0.925581395	2.79E-04	0.014059422	0.01405942
resveratrol	153	2	274	191	1715	0.961352657	3.43E-04	0.015909003	0.015909002
heart-mitochondria	154	1	297	172	1704	0.880530973	2.39E-04	0.014123658	0.014123655
amyloid-beta	155	4	259	165	1683	0.854077253	1.91E-04	0.014852404	0.014852402
accumulation	156	2	297	183	1676	0.925581395	2.90E-04	0.015682235	0.015682233
iron	157	4	297	182	1637	0.921296296	2.64E-04	0.016236239	0.016236238
rat-brain	158	4	267	177	1636	0.900452489	2.39E-04	0.01499481	0.014994808
vitamin-e	159	5	289	183	1629	0.925581395	3.18E-04	0.015015766	0.015015764
unfolded protein response	160	3	289	182	1621	0.921296296	3.17E-04	0.01525986	0.015259858
molecular-mechanisms	161	3	299	188	1613	0.947619048	3.36E-04	0.015992257	0.015992255
differentiation	162	3	294	177	1605	0.900452489	2.35E-04	0.016010628	0.016010627
cytochrome c	163	1	231	180	1602	0.912844037	2.68E-04	0.014980121	0.01498012
complex i	164	1	254	187	1596	0.943127962	3.04E-04	0.013937898	0.013937897
protection	165	5	273	180	1593	0.912844037	2.75E-04	0.014709225	0.014709224
transport	166	5	300	181	1591	0.917050691	2.73E-04	0.015138418	0.015138417
a-beta	167	4	261	158	1579	0.829166667	1.78E-04	0.014463639	0.014463637
parkin	168	2	250	162	1567	0.843220339	1.80E-04	0.013845447	0.013845445
reactive oxygen species (ros)	169	1	263	188	1539	0.947619048	3.17E-04	0.014218566	0.014218564
yeast	170	2	288	165	1513	0.854077253	2.00E-04	0.013815243	0.013815243
cyclosporine-a	171	1	256	162	1508	0.843220339	1.90E-04	0.013383886	0.013383883
longevity	172	2	262	166	1491	0.857758621	1.83E-04	0.013269485	0.013269484
rat-liver	173	1	285	177	1487	0.900452489	2.46E-04	0.014416328	0.014416326
mitochondrial membrane potential	174	1	235	188	1485	0.947619048	3.15E-04	0.012915961	0.012915959
reperfusion	175	1	223	172	1463	0.880530973	2.06E-04	0.012647319	0.012647318
ischemia-reperfusion	176	1	230	171	1462	0.876651982	2.13E-04	0.012856209	0.012856208
signaling pathway	177	3	259	178	1445	0.904545455	2.48E-04	0.014376738	0.014376736
cadmium	178	5	239	145	1443	0.786561265	1.02E-04	0.014096868	0.014096866
programmed cell-death	179	1	286	175	1424	0.892376682	2.56E-04	0.014192322	0.01419232
catalase	180	5	222	176	1410	0.896396396	2.33E-04	0.012423292	0.012423292
skeletal muscle	181	2	235	166	1406	0.857758621	1.78E-04	0.012941362	0.012941362
kidney	182	5	230	166	1404	0.857758621	1.99E-04	0.013913882	0.013913881
receptor	183	3	246	177	1381	0.900452489	2.47E-04	0.013302565	0.013302564
calorie restriction	184	2	247	169	1366	0.868995633	2.04E-04	0.01272523	0.012725229
therapy	185	3	260	177	1366	0.900452489	2.36E-04	0.013848948	0.013848947
ischemia/reperfusion injury	186	1	229	169	1363	0.868995633	2.12E-04	0.012537114	0.012537113
rat-liver mitochondria	187	1	259	167	1361	0.861471861	2.21E-04	0.012098474	0.012098472
oxidative-phosphorylation	188	1	311	184	1359	0.929906542	3.26E-04	0.008455747	0.008455749
transcription factor	189	2	264	176	1354	0.896396396	2.47E-04	0.012680125	0.012680124
glucose	190	2	233	179	1346	0.908675799	2.43E-04	0.012555725	0.012555724
age	191	2	232	177	1345	0.900452489	2.69E-04	0.012406814	0.012406813
oxygen species production	192	1	239	182	1343	0.921296296	3.02E-04	0.011958876	0.011958874
muscle	193	2	241	177	1342	0.900452489	2.32E-04	0.012271459	0.012271458
kinase	194	3	223	183	1331	0.925581395	2.71E-04	0.012397946	0.012397945
amyotrophic-lateral-sclerosis	195	4	253	162	1329	0.843220339	1.90E-04	0.012099103	0.012099102
mitochondrial dna	196	2	257	179	1310	0.908675799	2.64E-04	0.012285928	0.012285925
redox regulation	197	1	251	176	1310	0.896396396	2.40E-04	0.012567976	0.012567974
deficiency	198	2	246	178	1306	0.904545455	2.62E-04	0.012161376	0.012161375
substantia-nigra	199	4	215	148	1302	0.796	1.30E-04	0.011468685	0.011468683
transgenic mice	200	4	234	169	1268	0.868995633	2.30E-04	0.012117944	0.012117942
